# Scientific Rationale and Clinical Basis for Clindamycin Use in the Treatment of Dermatologic Disease

**DOI:** 10.3390/antibiotics13030270

**Published:** 2024-03-17

**Authors:** Maria K. Armillei, Ivan B. Lomakin, James Q. Del Rosso, Ayman Grada, Christopher G. Bunick

**Affiliations:** 1 Program in Translational Biomedicine, Yale School of Medicine, Yale University, New Haven, CT 06511, USA; maria.armillei@yale.edu; 2 Department of Dermatology, Yale University, New Haven, CT 06520, USA; ivan.lomakin@yale.edu; 3 College of Osteopathic Medicine, Touro University Nevada, Henderson, NV 89014, USA; jqdelrosso@yahoo.com; 4 JDR Dermatology Research, Las Vegas, NV 89148, USA; 5 Clinical Research and Strategic Development, Advanced Dermatology and Cosmetic Surgery, Maitland, FL 32751, USA; 6 Department of Dermatology, Case Western Reserve University School of Medicine, Cleveland, OH 44106, USA; ayman.grada@case.edu

**Keywords:** acne vulgaris therapy, antibiotic treatments, inflammatory skin disease, folliculitis, furunculosis, antimicrobial resistance, stewardship, skin and soft tissue infection

## Abstract

Clindamycin is a highly effective antibiotic of the lincosamide class. It has been widely used for decades to treat a range of skin and soft tissue infections in dermatology and medicine. Clindamycin is commonly prescribed for acne vulgaris, with current practice standards utilizing fixed-combination topicals containing clindamycin that prevent *Cutibacterium acnes* growth and reduce inflammation associated with acne lesion formation. Certain clinical presentations of folliculitis, rosacea, staphylococcal infections, and hidradenitis suppurativa are also responsive to clindamycin, demonstrating its suitability and versatility as a treatment option. This review describes the use of clindamycin in dermatological practice, the mechanism of protein synthesis inhibition by clindamycin at the level of the bacterial ribosome, and clindamycin’s anti-inflammatory properties with a focus on its ability to ameliorate inflammation in acne. A comparison of the dermatologic indications for similarly utilized antibiotics, like the tetracycline class antibiotics, is also presented. Finally, this review addresses both the trends and mechanisms for clindamycin and antibiotic resistance, as well as the current clinical evidence in support of the continued, targeted use of clindamycin in dermatology.

## 1. Introduction

Clindamycin is a widely studied and well-established antibiotic used in dermatological practice to treat a variety of skin conditions, including acne vulgaris. Clindamycin is classified as semi-synthetic and is part of the lincosamide family of antibiotics. It was isolated in the early 1960s from *Streptomyces lincolnensis* in Lincoln, Nebraska, and was synthesized via chemical derivatization of lincomycin from the original lincosamide species in 1966 in Kalamazoo, Michigan [1,2,3,4]. In 1970, the U.S. Food and Drug Administration (FDA) granted approval of clindamycin for clinical use [1,3].

Clindamycin has persisted as a viable therapy for over 50 years [1] and has a long-standing history of indications. Clindamycin was first used to treat various kinds of pneumococcal, staphylococcal, and streptococcal bacterial infections [3]. It acts as a potent and bacteriostatic agent against a number of Gram-positive aerobic bacteria, such as group A Streptococcus and several pneumococcal strains. Clindamycin is active against anaerobic Gram-positive and some Gram-negative genera, like Propionibacterium and Porphyromonas, respectively, and can also be used to treat severe streptococcal and staphylococcal infections, even in patients who have penicillin allergy [4,5,6]. Indeed, clindamycin may be used in cases of severe anaerobic bacterial infections of the respiratory tract, abdomen, and pelvic areas that are not responsive to penicillin [4]. Clindamycin can also be used to treat infections like cellulitis where cephalosporin- or penicillin-class antibiotic treatment may not be warranted [5,6]. Lastly, in certain cases, the antibiotic may also be effective against serious bacterial infections involving potentially resistant species like *Staphylococcus aureus* or *Staphylococcus epidermidis*, including *S. aureus* infections of the skin and soft tissues [4,5,6,7].

It is important to clarify that clindamycin is not truly a broad-spectrum antibiotic; instead, clindamycin has limited Gram-negative activity, as evidenced by its 133-fold higher minimum inhibitory concentration (MIC) against *Escherichia coli* (20 μg/mL) compared with *Staphylococcus aureus* (0.15 μg/mL). When oral clindamycin is ingested, it is absorbed during the first 45–60 min (an estimated 50–90% is absorbed via the gastrointestinal (GI) tract), at which time the maximum serum concentrations of clindamycin can be measured [4,6,8]. However, it has been estimated that anywhere from 0.01 to 30% of patients who are prescribed oral clindamycin may develop diarrhea concomitantly with treatment [4,9]. Additional complications, such as pseudomembranous colitis via *Clostridium difficile*, can range from mild to life-threatening. Cases of severe *C. difficile* are more likely to occur in hospitalized in-patients battling infections or with suppressed immune function [1,3,4]. Other, and less adverse, reactions may include GI distress, like nausea and vomiting, or skin hypersensitivity, such as rashes [9]. These disturbances are usually temporary and are often ameliorated after stopping antibiotic treatment, if necessary [8].

The challenge of antibiotic resistance, including to clindamycin, should not be ignored and will be detailed below [3,10,11]. Bacterial strains resistant to erythromycin can gradually adopt resistance to clindamycin [6]. Nevertheless, clindamycin is still a prime choice for the treatment of some anaerobic odontogenic infections orally or skin infections topically, including acne vulgaris [2,12]. While oral clindamycin can theoretically be used to treat acne [4], in practice, topical clindamycin has been a mainstay acne treatment in dermatology for many years. Topical antibiotics, including clindamycin, are often a primary treatment provided to acne patients depending on the severity of their acne [13].

Clindamycin exerts activity against *Cutibacterium acnes* [2]. *C. acnes* is an anaerobic, Gram-positive bacterium [2] and is a key player in acne pathogenesis, which includes events such as follicular hyperkeratinization, follicular rupture, and pro-inflammatory cytokine release [13,14,15]. Topical formulations of clindamycin alone, or those in combination with benzoyl peroxide (BPO to reduce antibiotic resistance as BPO is bactericidal), tretinoin, adapalene, or BPO plus adapalene (fixed-dose triple-combination gel clindamycin 1.2%/adapalene 0.15%/benzoyl peroxide 3.1%; IDP-126), have either been prescribed for many years or have recently been shown to be successful in clinical studies at managing inflammatory and non-inflammatory acne [2,13,16,17,18,19,20]. Importantly, clindamycin, when utilized in topical combinations to treat acne, confers minimal side effects, such as erythema or dry skin; such side effects should abate after acclimation to treatment, lowering the risk that patients will quit treatment [20]. Therefore, synergistic combination topicals containing clindamycin are effective treatments in dermatological practice. The continued use and novel formulation of topical therapies for acne that contain clindamycin is a prime example of why it is imperative and clinically relevant to discuss (i) the premise for the current use of clindamycin for a variety of dermatologic indications and (ii) the mechanism of action of clindamycin in the bacterial ribosome, with a special focus on its major target in acne, *C. acnes*. We must urgently and more precisely understand this mechanism of action given the frequency of topical clindamycin treatments prescribed. We must also understand (iii) the anti-inflammatory properties of clindamycin, (iv) trends in clindamycin use in relation to current antibiotic resistance patterns, and (v) the future of clindamycin use in dermatology.

For this review, the PubMed database was searched for peer-reviewed articles and clinical studies related to clindamycin and acne, using the initial following search terms: clindamycin, resistance, acne, minocycline, triple-combination therapy, mechanism of action, and ribosome. Several references in the articles from the initial search were also included and cited in this review. The aim of this review is to provide a greater understanding of how clindamycin is and has been used in dermatological practice, with emphasis on the current knowledge of its scientific mechanism of action reinforcing its most common and effective uses in dermatology.

## 2. Clindamycin Structural Mechanism of Action

### 2.1. Antibiotics Bind to Bacterial Ribosomes

The ribosome is the site of protein translation in the cell [21]. In eukaryotes, the ribosome consists of the 60S large and 40S small subunits; the 40S subunit reads the mRNA, while the 60S subunit enables elongation of the amino acid chain [22]. The mRNA contains codons, which are decoded via tRNA anticodons that match with individual mRNA codons; the placement of a new amino acid along the growing peptide chain results in peptide bond formation [21,22]. While bacterial ribosomes have some differences in composition compared with eukaryotic ones, they function very similarly overall to produce proteins. Importantly for medicine, bacterial ribosomes, but not eukaryotic ones, are target sites for antibiotics. For instance, in *E. coli*, the 70S bacterial ribosome is composed of the 50S large subunit and the 30S small subunit; the aminoacyl-tRNA site (A-site), peptidyl-tRNA site (P-site), and tRNA exit site (E-site) in the 30S subunit allow for mRNA decoding, and in the 50S subunit, they form the peptidyl transferase center (PTC) and the exit tunnel. After peptide bond formation, deacylated tRNA leaves the ribosome through the E-site, while the peptide chain is extended in the nascent peptide exit tunnel (NPET) in the 50S subunit [23,24]. Generally, the PTC and NPET permit the binding of commonly used antibiotics in dermatology to the 50S subunit of bacterial ribosomes; structural discrepancies in these ribosomal sites lead to selective antibiotic binding affinities and differential effectiveness based on the bacterial species and target site(s) of antibiotics within the ribosome [3,23,25].

### 2.2. Mechanism of Translation Inhibition by Clindamycin

**Figure 1:**
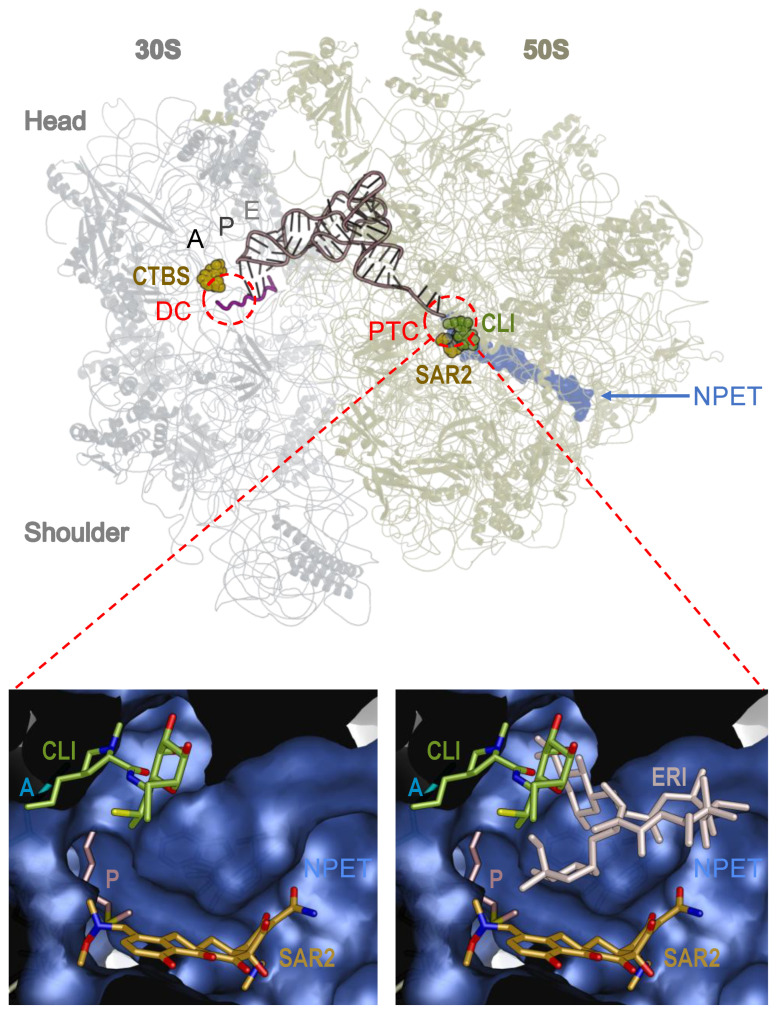
Interaction of Clindamycin with the 70S ribosome. Clindamycin (CLI, green), sarecycline (SAR2, gold) and erythromycin (ERI, beige) are shown as balls (top) or sticks (two bottom panels). Bottom panels are the surface representation of the NPET, with the clindamycin binding pocket depicted and CLI, SAR2, and ERI structures bound in their respective binding sites. P-site (P)-bound tRNA is shown in brown, A is the location of the A-site, and E denotes the E-site. The canonical tetracycline binding site is marked as CTBS, the decoding center as DC, the peptidyl transferase center as PTC, and the nascent peptide exit tunnel as NPET (blue). The 30S ribosomal subunit is in gray, 50S in khaki. Nitrogen atoms are blue, and oxygen atoms are red in CLI and SAR. Superposition of models with Protein Data Bank (PDB) IDs 8CRX, 4V7V, and 7NSO was used to create this figure [23,25,44]. All figures in this review were created using PyMOL Version 2.0 [45]. Abbreviations and labeling methods follow those presented in [23].

Clindamycin interacts with the PTC of the bacterial ribosome. In the PTC, clindamycin interferes with the proper orientation of the A- and P-site tRNAs, thereby inhibiting peptide bond formation and also sterically blocking progression of the nascent peptide (Figure 1) [26]. The macrolide class of antibiotics, whose binding site in the ribosome overlaps with that of clindamycin (lincosamide class), as well as for some tetracycline-class compounds (Figure 1), bind in the beginning of the NPET of the 50S ribosomal subunit close to the PTC and share the same mechanism of protein synthesis inhibition, despite structural differences [23,25,27].

**Figure 2:**
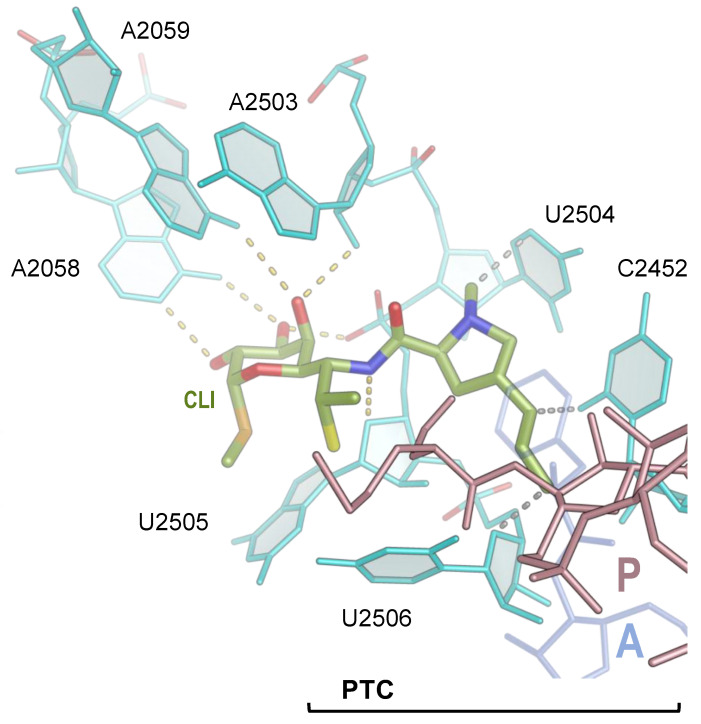
Clindamycin binding site in the 23S rRNA of the *E. coli* ribosome. Clindamycin (CLI, green) interacts with nucleotides of the 23S rRNA (cyan) as seen in the model of PDB ID 4V7V [25]. Hydrogen bonds are shown by yellow dashed lines, and van der Waals contacts are represented by gray dashed lines. To show the location of the aminoacyl moiety of the P-site (brown) and A-site (semitransparent blue) tRNAs in the PTC, the structure of the *E. coli* ribosome in PDB ID 7RQ8 [3] was superimposed. Nitrogen atoms are blue, and oxygen atoms are red in CLI.

The mechanism of protein synthesis inhibition by clindamycin was elucidated by crystallographic studies of its interaction with the large ribosomal subunit of G2058A *Haloarcula marismortui* and *Deinococcus radiodurans* and the 70S ribosome of *E. coli* [25,27,28]. The galactose sugar of clindamycin interacts with the nucleobases A2058 and A2059 (*E. coli* numbering here and below) and the sugars of A2503 and U2505, while its propyl pyrrolidinyl moiety interacts with the nucleobase of C2452 and U2504 and the sugar of U2506 of the 23S ribosomal RNA (rRNA) (Figure 2). However, it should be noted that the orientation of the propyl pyrrolidinyl moiety of clindamycin as well as the position of some other antibiotics were modeled differently in the *D. radiodurans* ribosome structure (discussed in [25,29]).

**Figure 3:**
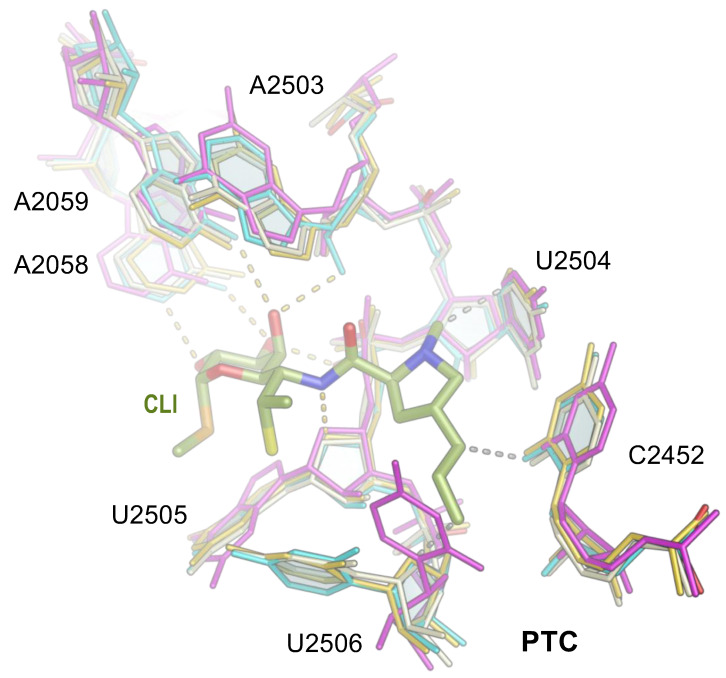
Clindamycin binding site in the 23S rRNA is conserved. The structure of the clindamycin (CLI) binding site (cyan, as shown in Figure 2) in the *E. coli* ribosome (cyan) was superimposed with that of *Thermus thermophilus* (gold, PDB ID 7RQ8 [3], *Staphylococcus aureus* (gray, PDB ID 5NRG [46]) and *Cutibacterium acnes* (magenta, PDB ID 8CRX [23]). Nucleotide U2506 is positioned differently in the *C. acnes* ribosome structure because this structure had no antibiotic bound to the PTC. This model suggests that the binding of clindamycin to *C. acnes* will induce a conformational change in U2506. Nitrogen atoms are blue, and oxygen atoms are red in CLI.

The sequence of rRNA forming the PTC and NPET is highly conserved (Figure 3), usually providing broader spectrum activity for antibiotics interacting with these regions of the ribosome. In addition to nucleotides in the binding site being important for the overall affinity of an antibiotic to the ribosome, it was also proposed that the nature of nucleotides in “the second shell” around the binding site may also affect antibiotic binding affinity [30,31]. For example, based on the structure of clindamycin bound to the *E. coli* ribosome, it was proposed that the alteration of C2055 in *E. coli* 23S rRNA—which stacks on U2504 (Figure 2)—to A2055 in the 23S rRNA of archaeon *H. marismortui* would displace four universally conserved nucleotides U2504, G2505, U2506, and C2507, thus reducing hydrogen bonding with clindamycin and providing an explanation as to why archaeal and eukaryotic ribosomes bind lincosamides poorly [25]. It is not surprising, then, that mutations of the nucleotide 2504 increase the minimum inhibitory concentration (MIC) of clindamycin [32].

### 2.3. Clindamycin Function in C. acnes

*C. acnes* is the major bacterial species that is involved in the pathogenesis of acne [2,13] and can be targeted by antibiotics including clindamycin. For other dermatologic indications, such as hidradenitis suppurativa (HS) and rosacea, clindamycin may be prescribed in some mild cases; however, primary pathogenic bacteria linked to disease etiology and solely targetable by clindamycin as a monotherapy for such conditions have not been described [33,34,35,36]. Clindamycin halts the growth of the anaerobic, Gram-positive bacterium *C. acnes* by inhibiting protein translation through binding to the 50S subunit of *C. acnes* to prevent peptidyl tRNA translocation within the *C. acnes* ribosome and formation of the peptide bond [1,2,4,7,20,37]. There is no experimental structure to date of clindamycin bound to the *C. acnes* ribosome; however, it is surmised that clindamycin functions similarly in *C. acnes* as it does in *E. coli*. Clindamycin binds to the 23S rRNA of the large 50S bacterial ribosomal subunit at the PTC site, with additional proposed effects on aminoacyl-tRNA binding with the A-site [25,38], albeit near the NPET entrance (Figure 1). Similarly, erythromycin, a macrolide, binds to the 50S subunit at the 23S rRNA [2,37,39] of the NPET [40,41], which prevents the exit of any synthesized peptide from entering the intracellular space [25]. Of note, antibiotic resistance to clindamycin and macrolides are prominently conferred via 23S rRNA nucleotides [25]. Clindamycin performs hydrogen bonding with 23S rRNA residues, including A2058, A2059, A2503, U2506, G2505, and C2452, within the 50S subunit [38]. Conversely, tetracyclines target both Gram-negative and Gram-positive bacterial species, including *C. acnes*; they inhibit protein translation by binding at the 16S rRNA site of the 30S bacterial ribosomal subunit [42,43]. Tetracycline binding to the 30S subunit interrupts the inflow of the incoming tRNAs to the A-site to effectively diminish protein translation within the *C. acnes* ribosome [24,42] (Table 1).

## 3. Efficacy and Use of Clindamycin in Dermatologic Disease

### 3.1. Acne Vulgaris

#### 3.1.1. Clindamycin Therapy Targets *C. acnes*

Clindamycin in dermatological practice is largely used to treat acne vulgaris. It can also be used to treat bacterial folliculitis, rosacea, mild hidradenitis suppurativa, and other Gram-positive cutaneous infections. First, acne occurs in more than 85% of people between 12 and 24 years of age and is prevalent in nearly 10% of the global population [17]; it may continue as a chronic condition in patients for up to 30 years [20], or at least well into adulthood, if left untreated. *C. acnes* is the target bacterium in acne [2]. Prior to 2016, *C. acnes* was formally called *Propionibacterium acnes*, or *P. acnes*; the reclassification to *C. acnes* was based on updated sequencing, taxonomic classification techniques, and a greater understanding of the human microbiome and skin flora, which have together enabled the determination of *C. acnes* phylotypes and other characteristics of the bacterium [47,48]. This review denotes the bacterial species as *C. acnes*, although the prior term *P. acnes* was used in some of the research articles included in this review.

Research utilizing sequencing approaches, such as metagenomic shotgun sequencing and other next-generation sequencing (NGS) capabilities, suggested that the hyperproliferation of *C. acnes* is not the sole cause of acne [47,48]. Rather, specific ribotypes (i.e., RT4 and RT5) of the putative pathogenic phylotype IA of *C. acnes* may be to blame, thus implicating specific *C. acnes* strains in relation to factors like virulence, biofilm formation, and inflammation that are involved in acne pathogenesis [47,48]. While the composition of the skin flora may be an influential but not the main causative factor in acne lesion development [48,49], the findings that *C. acnes* forms colonies and biofilms in the pilosebaceous units of acne-prone skin demonstrates that *C. acnes* has a prominent role in the pathogenesis of acne, including in inflammatory acne [48,50].

#### 3.1.2. Evidence in Favor of Standard and Novel Topical Clindamycin Treatments for Acne

Clindamycin has been used for more than 30 years in dermatology, rendering it a highly effective and safe treatment option for acne [2]. Clindamycin in topical form is found in formulations concentrated at 1–2% of the total vehicle cream, lotion, foam, gel, etc., and is widely manufactured in topical products with other prescription-strength actives like BPO [2,37,51]. Side effects of topical clindamycin may include skin peeling, erythema, burning, and dryness in the treatment area [2,15]. Allergic dermatitis may also occur with topical clindamycin treatment, although fewer than 20 cases have been discussed in the literature [52]. Clindamycin possesses antibacterial and anti-inflammatory properties that are thought to work in a synergistic fashion to diminish *C. acnes* growth and reduce inflammation, respectively [2]. Therefore, blunting the activity of *C. acnes* with clindamycin may diminish the bacterial growth and inflammatory havoc that ensues from *C. acnes* colonization in acne-prone skin [2]. For instance, by comparing the antibacterial activity of clindamycin with that of erythromycin, one study [53] observed the inhibition of *C. acnes* growth was greater using a clindamycin phosphate lotion in acne patients than when tetracycline or erythromycin topical solutions were administered in parallel to the clindamycin topical [2]. Another study [54] found that the effects on acne lesion counts in moderate to severe acne patients were similar between patient groups that received a clindamycin topical (clindamycin phosphate 1%) versus those that received oral minocycline (50 mg of minocycline). Thus, for some patients, treatment with a clindamycin topical is a suitable alternative to oral antibiotics [2,54].

Clindamycin has a high strength of recommendation for treating acne in recent guidelines, alongside other topical antibiotics such as erythromycin and topical retinoids like adapalene and tretinoin [55]. International guidelines suggest the use of BPO with either clindamycin or erythromycin in order to treat comedonal acne (i.e., whiteheads and blackheads); meanwhile, treatment combinations of clindamycin and tretinoin may be used for more severe acne cases that involve papulopustular lesions [35]. Topical clindamycin is generally preferred over erythromycin owing to the greater chance that patients will become resistant to erythromycin; the avoidance of clindamycin in a topical solution by itself is also suggested [13]. As with most topical treatments, patients must allow at least 12 weeks to see results from using a clindamycin topical [13].

BPO is a common topical bactericidal treatment option for acne and is available over-the-counter (OTC) in a variety of 2.5%, 5%, and 10% formulations; BPO can decrease both acne inflammatory lesion count and the extent of lipolysis of triglycerides that are contained in sebum [2,15]. Notably, BPO does not contribute to antibiotic resistance, and it functions efficaciously in dual combination with topical antibiotics like clindamycin (i.e., BPO 5%, clindamycin 1% gel or lotion); clindamycin can also be found in combination with adapalene or tretinoin (i.e., clindamycin phosphate 1.2%, tretinoin 0.025% gel) [2]. The use of clindamycin in fixed-combination formulations minimizes the chance of bacterial resistance, which is known to result from the use of just one topical antibiotic alone in a treatment regimen [56].

Topical clindamycin/BPO combination treatments have been shown in several clinical trials to reduce total acne lesion counts in patients to a greater degree than either clindamycin or BPO treatment alone [20]. For instance, a clindamycin 1%, BPO 5% gel was well tolerated in patients utilizing twice-daily applications of the topical; the patient discontinuation rate of treatment over a period of 10–16 weeks was only between 0 and 0.8% [20]. Topicals containing either clindamycin with BPO (i.e., clindamycin 1%, BPO 5% gel) or BPO with adapalene (i.e., 2.5% BPO, 0.1% adapalene gel) are usually sought after as primary treatments in the management of acne that is predominantly on the face [57]. While both types of treatments may be similarly tolerable, results from a split-face treatment study of 2 weeks [57] demonstrated that there was greater cutaneous tolerability and satisfaction after using a clindamycin 1%, BPO 5% topical versus in response to using a 2.5% BPO, 0.1% adapalene combination gel. However, in one 12-week trial [58], no significant differences in the reduction of non-inflammatory and inflammatory acne lesion counts were found between treatment groups that received either a clindamycin 1%, BPO 5% topical or an adapalene 0.1%, BPO 2.5% gel. Moreover, another study [59] found that a clindamycin phosphate 1.2%, BPO 2.5% fixed-combination gel was effective for moderate-to-severe acne and was tolerated with minimal side effects in a treatment population of adolescents with skin of color. The tolerability of clindamycin/BPO topical formulations in treating acne demonstrates that clindamycin topicals are effective and safe and may even be used alongside concurrent acne treatment methods [20].

Notably, a 12-week, multicenter study utilizing the first triple-combination acne product containing clindamycin phosphate 1.2%, BPO 3.1%, and adapalene 0.15% (IDP-126) was found to significantly reduce inflammatory and non-inflammatory acne lesion counts (resulting in greater than 70% reductions) in a treatment population of 741 patients with moderate-to-severe acne [13,19]. A phase II study [19] demonstrated not only the efficacy of the triple-combination therapy for acne, but also the effectiveness of IDP-126 over vehicle and dyad gels, such as a clindamycin phosphate/BPO gel, on the evaluated treatment measures, like tolerability and inflammatory and non-inflammatory acne lesion reduction. Another study [16] described the efficacy of a fixed-combination clindamycin phosphate 1.2% plus tretinoin 0.025% gel that, when applied once daily, was safe and effective in treating acne across a range of severity.

#### 3.1.3. Clindamycin Is an Effective Treatment across Acne Patient Populations

Clindamycin combination topicals are also relevant for the treatment of perimenstrual—commonly known as hormonal—acne in females; flare-ups of premenstrual acne, and the development of inflammatory acne lesions associated with the latter portion of the luteal phase, may affect just under 50% or more than 60% of women, respectively [18]. A pilot study of this nature [18] established that the once-daily application of a clindamycin phosphate 1.2%, BPO 3.75% gel formulation significantly reduced acne lesion counts in relation to self-reported perimenstrual acne. Local cutaneous reactions, such as dry skin and erythema, were generally mild and resolved over the course of the 12-week treatment [18]. Therefore, the potential for fixed-combination clindamycin-containing topicals to have success against hormonal acne is a win—largely because these treatments can prevent *C. acnes* from flourishing in an otherwise androgen-, keratin-, and sebum-rich environment, which generally characterizes the pathophysiology of hormonal acne [18] along with inflammation.

Of note, pediatric patients seek dermatologic care more often for acne than for any other dermatologic condition [60]. For patients in this age group, referring primary care pediatricians are not as likely to prescribe oral antibiotics to treat acne as they are to suggest OTC BPO for acne management; similarly, dermatologists may first suggest or prescribe BPO as a primary acne treatment for pediatric patients [60]. Importantly, acne can be a chronic, disfiguring skin condition that is an increasingly common mental health burden on people with this condition. Physicians should continue to bear in mind the psychosocial factors and the wellbeing of acne patients in offering safe and effective treatment options [61]. Acne topicals versus systemic treatments may be effective options for a younger population. For example, adolescents with acne have a preference for topical treatments, and clindamycin phosphate 1.2%, BPO 3.75% topical gel significantly reduced non-inflammatory and inflammatory acne lesion counts in patients 12 to less than 18 years of age [62]. Thus, clindamycin-containing topicals are viable treatment options for patients of all ages and skin types across a range of acne lesion types and severity.

### 3.2. Folliculitis

#### 3.2.1. Bacterial Folliculitis

Clindamycin can be used to treat certain types of folliculitis, a common skin condition that occurs when hair follicles become infected [63]. Folliculitis may present either as a superficial infection or an infection of the deep layer of the hair follicle, and it results in the formation of papules or pustules over the area of the inflamed follicle [63]. Bacterial folliculitis tends to present as papules and pustules that are monomorphic and folliculocentric in nature, without the formation of comedones; this type of folliculitis may initiate from the presence of *S. aureus* colonization within the hair follicle [15,63]. Superficial bacterial folliculitis can be treated with the application of topical clindamycin, such as clindamycin phosphate 1% foam [64]. While folliculitis is generally bacterial in nature, viruses or fungal species can also instigate other folliculitis types and their spread. Importantly, not all cases of folliculitis are infectious, such as when folliculitis ensues from inflammation resulting from ingrown hairs [63]. Gram-negative bacterial folliculitis is commonly caused by the Gram-negative bacterium *Pseudomonas aeruginosa*; the overuse of certain oral antibiotics can foster Gram-negative bacterial folliculitis in certain individuals [63].

#### 3.2.2. Fungal Folliculitis

*Pityrosporum* folliculitis, also known as *Malassezia* folliculitis, is a type of fungal folliculitis initiated by the *Malassezia* fungal species in the skin [63,65]. *Malassezia* folliculitis presents in a similar manner to bacterial folliculitis, but with the addition of pruritic papules and pustules that tend to appear on the upper portion of the back, hairline, chest, and even the face [15,65]. *Malassezia* folliculitis can notoriously present like acne and may frequently be treated as such (i.e., by following a standard acne treatment regimen of oral antibiotics) in the clinic to no avail and with inadequate clearance of symptoms [65]. Oral antibiotic use may perpetuate *Malassezia* yeast overgrowth, possibly by altering the skin flora, which can inadvertently complicate *Malassezia* folliculitis [65]. However, acne and *Malassezia* folliculitis can also coexist (in up to 27% of cases) [65]. Generally, *Malassezia* folliculitis treatment involves the administration of oral or topical antifungals, like ketoconazole, while acne medications, albeit not antibiotics, may also be required to minimize the presence and number of acne lesions [65]. The mechanisms of action of oral and topical antibiotics on *C. acnes* inhibition make it clear that antibiotics like clindamycin work especially well for acne treatment, while other treatments, such as antifungals, may work better for treating cases of folliculitis where the infectious species that predominates is fungi instead of bacteria.

#### 3.2.3. Other Folliculitis Types

Some antibiotics commonly used to treat acne can also be used in the treatment of other, less common, folliculitis types. Viral folliculitis has a similar appearance on the skin to bacterial folliculitis, but instead, the agent of infection is usually herpes virus [63]. Viral folliculitis normally presents in clustered plaques on the skin, in contrast to the individual pustules that are seen with bacterial folliculitis [63]. *Demodex* folliculitis is caused by the *Demodex folliculorum* mite. Eosinophilic folliculitis can arise in individuals with HIV infection and can present as pustules [63].

Lastly, folliculitis decalvans (FD) is a rare type of folliculitis, primarily causing cicatricial alopecia [66]. This type of folliculitis is neutrophilic in nature and is thought to stem from a disrupted immunological response in combination with the presence of *S. aureus* within the hair follicles of the skin; the younger the age of onset of the condition, the more severe FD symptoms tend to be [66]. Clinical presentation of FD on the scalp can include hyperkeratosis and alopecic patches, as well as tufted hairs, where greater than 5 hairs grow out of a follicular orifice; these manifestations can be associated with inflammation and alopecic scarring [66]. Over a range of severity of FD cases, different treatment options include: the use of topical steroids and antibiotics, oral tetracyclines, a combination of clindamycin and rifampin, and/or oral isotretinoin or systemic steroids [66]. Clindamycin and rifampin treatment was demonstrated to be effective in a case study of relapsing FD, where 300 mg of both oral clindamycin and rifampin reduced signs of inflammation and alopecic scarring over 10 weeks of treatment [66].

### 3.3. Rosacea

Rosacea is a common inflammatory skin condition. It generally presents as erythema, flushing, and the formation of papules or pustules on the face [35,67]. Ocular symptoms such as eye redness and dryness are also common and can occur in up to 75% of rosacea patients [67]. The four main subcategories of rosacea are phymatous rosacea, ocular rosacea, papulopustular rosacea (PPR), and erythematotelangiectatic rosacea (ETR), and patients may present with more than one type of rosacea [67,68]. While the complete pathophysiology of rosacea has not been fully elucidated, environmental factors such as UV exposure, as well as alcohol intake, altered innate and adaptive immunity, and genetics, may exacerbate rosacea and its associated symptoms [35,67,68]. The presence of *Demodex folliculorum* (mites) on the skin and *Helicobacter pylori* infection in the GI tract may also be involved in rosacea—notably, disorders of the GI tract, such as Crohn’s disease, ulcerative colitis, and irritable bowel syndrome (IBS), among others, have been reported to affect the cutaneous microbiome via a dysregulated immune system and may be connected with rosacea presentation [67,68]. Regarding bacterial species involved in rosacea, the Gram-negative bacterium *Bacillus oleronius* has been associated with rosacea [67,68]. *S. epidermidis* is also associated with rosacea pustules, and interestingly, a reduction in *C. acnes* colonization has been found in rosacea facial skin, especially in PPR and ETR [68].

Treatment regimens for rosacea aim to mitigate both cutaneous inflammation in the skin as well as the presence of any papular and/or pustular lesions. While the treatment of potential bacterial species involved in rosacea, such as *Bacillus oleronius*, may be a target of interest, antibiotics like oral tetracyclines are largely reserved for refractory treatment as well as symptoms that do not respond to primary topical antiparasitics, topical antifungals, and topical antibiotics [68]. Rosacea treatment commonly involves the administration of topical metronidazole, azelaic acid, and/or ivermectin. Metronidazole and ivermectin can be used as complementary therapies for the treatment of papules and pustules in PPR [36,68]. The administration of metronidazole (0.75% or 1%), along with its anti-inflammatory modalities in the skin, make it a widely accepted topical treatment alone, especially for many cases of PPR and erythematous rosacea [35,36]. Treatments for PPR may consist of both oral and topical therapies, such as an oral tetracycline (minocycline, doxycycline, or sarecycline) and topical clindamycin (i.e., clindamycin 1% gel or clindamycin 1%/BPO 5% gel) in a coadministration regimen, respectively, to reduce PPR-associated inflammation [36,69]. While rosacea symptoms may be ameliorated with some antibiotic treatment regimens, because a bacterial infection is not thought to be the root cause of rosacea pathophysiology, treatment with topical and oral antibiotics may not provide total symptom resolution when used as the sole treatment regimen [35].

### 3.4. Hidradenitis Suppurativa (HS)

Clindamycin can also be used to treat hidradenitis suppurativa (HS), also known as acne inversa [34]. HS presents as inflammatory lesions within the follicular epithelium or sweat glands in intertriginous areas of the body like the axillae, buttocks, inframammary, and medial thighs [33,34,35]. HS more often affects adult women who have obesity and who smoke and is thought to involve autoimmunity linked with environmental components and metabolic syndrome pathophysiology [34,35]. Despite the fact that HS is not a purely infectious disorder, clindamycin (i.e., a clindamycin 1% topical) is a recommended option for the treatment of mild (Hurley stage I) HS (i.e., pustular lesions on the surface of the skin and no abscesses or sinus tracts) [35]. Similarly, clindamycin and dapsone are an effective treatment combination for mild HS, yet for more serious HS cases (Hurley stages II and III), a regimen of oral minocycline, doxycycline, or clindamycin along with rifampin may be suitable [33]. In treating moderate to severe HS, the current standard practice is to administer biologics [70]. Thus, we note more advanced HS may require biologic therapy, either FDA-approved adalimumab or secukinumab; however, this review will focus on clindamycin and other antibiotic therapies. A major reason for using clindamycin to treat HS stems from the fact clindamycin can be an effective antimicrobial against staphylococcal species and can curb inflammation [33,35].

### 3.5. Staphylococcal Infections

Clindamycin has historically been used to treat a range of infections, spanning from superficial infections to deeper infections of soft tissues [8]. At its inception, clindamycin was used for bacterial infections involving staphylococcal, pneumococcal, and streptococcal species [3,6]. Patients with severe bacterial infections (such as severe staphylococcal infections) and those who are allergic to penicillin may be prescribed clindamycin for such infections; importantly, clindamycin is reportedly efficacious when used to treat some methicillin-resistant *S. aureus* or MRSA infections [4,5,6,8]. However, caution is advised when prescribing clindamycin for MRSA, as methicillin-resistant *S. aureus* strains may also be clindamycin-resistant [6]. Clindamycin can also be effective in treating some *S. epidermidis* infections, but species resistance to clindamycin should be determined via sensitivity testing before administration of the antibiotic [4]. Caution must be taken when prescribing oral or systemic clindamycin; contraindications include colitis, like ulcerative colitis, or a patient history of sensitivity to clindamycin [8]. Nevertheless, clindamycin is an effective, relatively low-cost treatment and is readily available to physicians to treat many types of bacterial infections [8,11]. Ultimately, clindamycin exerts strong activity against a range of infections, from mild skin infections to more serious infections of deeper tissues (Table 2).

### 3.6. Additional Indications and Contraindications

Broadly, clindamycin has been FDA-approved for the treatment of a range of abdominal, bone, joint, pelvic, respiratory, and gynecologic (i.e., bacterial vaginosis) infections [6,8]. Severe pneumonia in hospitalized in-patients may warrant the administration of intravenous clindamycin as treatment [8]. Clindamycin is an alternative treatment for impetigo and abscesses of the skin [6]. Clindamycin may also be used as a prophylaxis treatment prior to dental or surgical procedures or surgical anesthesia; it also can be utilized alongside gentamycin, with or without ampicillin, in the treatment of endometriosis in certain instances [8]. Also, clindamycin is effective against streptococcal myositis, necrotizing fasciitis, and cellulitis as it can quickly penetrate into underlying soft tissues [11,71]. Some babesiosis, malaria, and anthrax infections may also be treated with clindamycin [8]. Thus, clindamycin can be used to treat a wide range of infections caused by many bacterial species [6].

Additional dermatologic indications for which clindamycin is a possible treatment option include: pitted keratolysis, cutaneous erythrasma, progressive macular hypomelanosis, and perioral dermatitis [72,73,74,75]. Clindamycin may be used “off-label” for these and some other indications, provided at least some available clinical findings back the use of clindamycin for such conditions. Furthermore, prescribing clindamycin in dermatologic practice is largely influenced by (i) oral or topical administration and (ii) patient history, with the primary goals of safety and efficacy. For instance, oral clindamycin is not advised for patients sensitive or allergic to clindamycin/lincosamides, and indeed, it is contraindicated in patients who have experienced ulcerative or pseudomembranous colitis (antibiotic-associated colitis) [8]. Caution must be taken when intending to treat patients with clindamycin who have liver disease [8]. Sensitivity to topical clindamycin may result in an adverse reaction of maculopapular eruptions; more severe reactions (i.e., acute generalized exanthematous pustulosis (AGEP), Stevens-Johnson syndrome, and drug rash with eosinophilia and systemic symptoms (DRESS)) to topical clindamycin are not common [8,76,77,78,79,80,81]. However, such adverse reactions may be managed by stopping administration of the antibiotic and delivering fluids and corticosteroids [8].

Finally, in special populations, i.e., in persons pregnant or lactating, it is not advisable to administer clindamycin. Concerning oral administration, unless the necessity for its use outweighs the risks, oral clindamycin in the long term is cautioned against and topical clindamycin (i.e., for acne treatment) is not suggested as (i) clindamycin can be found in breast milk and (ii) safety considerations of clindamycin for dermatologic use when pregnant or breastfeeding have not been holistically described [71,77,82,83]. Overall, it is pertinent to acknowledge that clindamycin is a suitable treatment option for a range of dermatologic indications, but also that safety, efficacy, and individual patient histories must be considered when prescribing clindamycin in accordance with evidence based medicine.

**Table 2:** Summary of the use of clindamycin for several dermatologic indications.

Skin Condition	Clindamycin Treatment Type	References
Acne vulgaris (common acne)	Topical clindamycin phosphate (i.e., clindamycin phosphate 1%), though clindamycin monotherapy is not recommended	[2,13,15,16,17,18,19,35,54,57,59,62]
	Topical clindamycin in combination: Clindamycin/BPO (i.e., 1%/5%; or fixed-combination 1.2%/2.5% or 1.2%/3.75%) Clindamycin/Tretinoin (i.e., 1%/0.025% or 1.2%/0.025% gel)	
	Topical clindamycin in triple-combination (IDP-126): clindamycin phosphate/BPO/adapalene (1.2%/3.1%/0.15%)	
	Adapalene/BPO (i.e., 0.1% or 0.3%/2.5%)	
	BPO alone (2.5–10%)	
Folliculitis (i.e., bacterial superficial, bacterial Gram-negative, *Malassezia* (*Pityrosporum*) folliculitis, folliculitis decalvans (FD))	For common bacterial/superficial folliculitis: clindamycin phosphate (1%) topical foam, solution, or gel	[64,65,66,84]
	For Gram-negative bacterial folliculitis or pustular acne: ampicillin or topical gentamycin and oral co-trimoxazole, followed by clindamycin/BPO/tretinoin and/or oral tetracyclines	
	For severe/refractory FD: clindamycin/rifampicin treatment (i.e., 300 mg/each, 1× day/10 weeks) or oral isotretinoin or steroids; for mild FD: topical corticosteroids or topical antibiotics (2–3×/week)	
	For *Malassezia folliculitis*: oral antifungals, like ketoconazole	
Rosacea, including papulopustular rosacea (PPR)	Sole treatment or effective combination of: metronidazole (0.75%, 1%), ivermectin (1%), azithromycin (2%), azelaic acid (15%, 20%), erythromycin (2%)	[35,36,68,85]
	PPR treatment for more severe cases may also include: oral doxycycline/minocycline/sarecycline and topical clindamycin (1%) gel or clindamycin/BPO (1%/5%) gel	
Staphylococcal infections	For *S. aureus*: at least 0.1 μg/mL minimum inhibitory concentration (MIC) clindamycin	[6,86]
	Severe: 7 days of 10 mg/kg/dose clindamycin as an antitoxin adjunct treatment may be administered intravenously (IV)	
Hidradenitis suppurativa (HS)	For mild HS, generally topical clindamycin (1%), or topical clindamycin/dapsone combination	[33,35]

## 4. Review of the Anti-Inflammatory Properties of Clindamycin

Strong evidence suggests that clindamycin possesses significant anti-inflammatory properties within the skin in addition to its antibacterial activity [2,15,20,87]. While the anti-inflammatory mechanisms of clindamycin are not fully understood, the ability of clindamycin to reduce both bacterial growth and inflammation associated with a range of dermatologic conditions, especially acne, is increasingly recognized [10,14,61,88,89,90]. A better understanding of the anti-inflammatory properties of clindamycin may lead to the development of new dermatologic treatments. It is also necessary to distinguish between the antibacterial and anti-inflammatory properties of clindamycin. The following sections dissect the anti-inflammatory characteristics of clindamycin, with an emphasis on its use in ameliorating inflammation in acne, in accordance with current evidence. It is also important to bear in mind that key inflammatory signals (e.g., Th1/Th17 response) may be common not only to acne, but to other indications as well (i.e., rosacea, HS); insights into specific inflammatory processes for the latter will be presented here and have been reviewed in great detail elsewhere [33,34,35,36,67,68,70,85].

### 4.1. Crosstalk between Clindamycin and the Immune System in Acne

Clindamycin prevents the production of chemotactic factors that arise from polymorphonuclear leukocyte (PMN) cell activation and signaling in chemotaxis [2,20]. Interestingly, the anti-inflammatory modalities of clindamycin may hamper PMN chemotaxis itself, as clindamycin can even permeate into PMNs [9]. These factors illustrate the interaction between clindamycin and innate immune factors within the pilosebaceous unit [14] (containing the hair follicle, arrector pili muscle, and sebaceous gland) [91]. For instance, PMNs release pro-inflammatory hydrolytic enzymes that can exacerbate inflammation in the pilosebaceous unit [20]. Clindamycin may also downregulate the expression of toll-like receptor (TLR)-2 that gives rise to the upregulated expression levels of Th1 and Th17 T helper cells; notably, the activities of these cells are also upregulated in papulopustular rosacea (PPR) [36,68,87]. TLRs like TLR-2 can be found in macrophages and keratinocytes within the follicle and can contribute to acne pathogenesis [11,14,61]; TLR-4 activity may also contribute to acne pathophysiology [89]. The presence of *C. acnes* can also activate TLRs and protease-activated receptors (PARs) in keratinocytes, leading to the release of antimicrobial peptides (AMPs) from keratinocytes and sebocytes in the sebaceous gland [11,14]. Thus, adaptive and innate immune system activities are involved in inflammation during acne pathogenesis [15].

The development of inflammatory acne lesions may result from a change from skin homeostasis to a state of inflammation [87], a process which is thought to involve: (i) excess sebum production, (ii) increased *C. acnes* presence and metabolic activity, leading to cytotoxic metabolite release in the pilosebaceous unit, (iii) pro-inflammatory cytokine release, (iv) recruitment of immune cells to the acne lesion locale, (v) incessant *C. acnes* pathogenic activity marked by biofilm formation and immune cell involvement, and (vi) an overall alteration to and dysbiosis of the skin flora in acne patients [14,15,87,92,93]. The succession of events outlined above remains a debated topic; for example, immune cells may first initiate hyperkeratinization and comedone formation [61,87,94]. The presence of *C. acnes* in the pilosebaceous unit may also trigger immune responses prior to hyperkeratinization [15]. Importantly, hyperkeratinization is a hallmark of acne pathogenesis: increased expression levels of filaggrin and IL-1α [95,96], amidst the excess proliferation of keratins 6, 16, and 17, are reportedly involved in this process [89,97]. Deviations from normal keratinocyte differentiation processes and alterations to the composition of lipids within the sebaceous gland may arise from incoming hormonal signals and their effects on the release of inflammatory cytokines like IL-1 [61]. Immune cell activity within the follicle may also influence *C. acnes* overgrowth and additional TLR-2 stimulation, Th1 activity, and pro-inflammatory cytokine release [89]. Signaling by CD3+ and CD4+ T cells in the pilosebaceous unit may also occur prior to hyperkeratinization of the follicle [61,90].

### 4.2. Inflammation Mediated by C. acnes in Acne Pathogenesis

#### 4.2.1. Role of Inflammation in Acne Lesion Formation

Questions remain as to whether acne lesion formation is inflammatory in nature at its inception; acne etiology involves accruing sebaceous gland density especially on the face and after the onset of puberty, which gives rise to a simultaneous increase in androgen hormone production and heightened androgen receptor activity in the skin [15,18,61,87]. Increased androgen signaling is thought to initiate hyperkeratinization and greater sebum production [18]; thereafter, the formation of a microcomedo, a change to skin homeostasis [87], and immune cell involvement may underlie acne lesion development [15].

A challenge to the conventionally proposed role of inflammation in acne is the argument that immune responses are involved not only in the formative stages of inflammatory acne lesion development, but also throughout the entirety of acne pathogenesis and perhaps even prior to the growth of comedones into inflammatory papules or pustules [90]. This idea challenges the belief that *C. acnes* colonization in the follicle is what causes the inflammatory shift from comedonal to inflammatory acne and that only papular, pustular, and nodular lesions are those that are inflammatory in nature [90]. Instead, the possibility exists that innate and adaptive immune system activities in the pilosebaceous unit may arise before *C. acnes*-induced inflammatory signaling; thus, the mechanisms for immune system regulation in the comedonal to inflammatory acne lesion switch, or in the process of comedone formation itself, requires further research [90].

Returning to the primary argument that inflammatory acne lesions develop after a comedonal switch to an inflammatory state, it is thought that the growth of a microcomedo occurs prior to comedone formation and comedonal acne development [15]. Comedones that are closed are called whiteheads and open comedones are blackheads; whiteheads are marked by their papular shape, whitish dome top, and do not possess clinical inflammatory signs, while blackheads have a black color in appearance owing to the presence of a keratotic plug and melanin that has been oxidized at the comedone opening at the skin surface [15,61]. Comedonal acne lesions (comedones) constitute non-inflammatory acne, while inflammatory acne consists of papules and pustules, as well as nodulocystic lesions in severe acne [15,61].

#### 4.2.2. *C. acnes*, Sebum, and Antimicrobial Peptides (AMPs)

The interaction between *C. acnes* and sebum also contributes to inflammatory acne lesion formation. Excess sebum and the abundance of *C. acnes* in the pilosebaceous unit may shift the type of acne lesion from comedonal to inflammatory; this switch may occur in part due to the production of lipase and the metabolism of triglycerides into their fatty acid (FA) and glycerol components by *C. acnes* within the sebaceous gland [15,61]. The action of free FA breakdown in the follicle, followed by the release of pro-inflammatory cytokines (e.g., IL-1, IL-8, and IL-12) and the activity of AMPs like defensins (i.e., defensin-2) [90], as well as increased levels of serum calprotectin [98], are all factors reportedly involved in perifollicular inflammation and the emergence of inflammatory acne lesions [15,61,99,100,101,102].

*C. acnes* is a known commensal bacterium on human skin, yet its involvement in the pathogenesis of acne has not been fully elucidated. Certain virulent and acne-causing strains of *C. acnes* are thought to be promoters of inflammation [10,14,15,92]. Building upon the idea that *C. acnes* activity within the pilosebaceous unit is central to inflammation in acne [90], it has been reported that the pilosebaceous unit is a hostile, oxygen-poor environment [92], but that *C. acnes* thrives in this environment by feeding on sebaceous lipids; thus, sebum quantity is likely directly related to *C. acnes* colonization on the skin and acne severity [61]. Also, sebaceous glands are inherently key players in the innate immune system and have endocrine system-responsive properties; while they contribute to the production of sebum, they also secrete AMPs [61]. Although one key aspect of clindamycin in the treatment of acne is that the antibiotic can halt the growth of *C. acnes*, it has also been proposed that AMPs, which are antibacterial agents produced by the body itself, can likewise modulate both the growth of *C. acnes* and cytokine release [88]. Future research on AMPs and their antimicrobial activity in relation to that of antibiotics like clindamycin is needed to determine how, and if, AMPs aid or hinder pro-inflammatory processes in the development of acne lesions, and what their specific interactions are with *C. acnes* in acne pathogenesis [88].

#### 4.2.3. *C. acnes* Phylotypes and Acne Severity

It has been hypothesized that a disruption to the homeostatic skin equilibrium that is composed of diverse bacterial species, including *C. acnes* strains, combined with cutaneous immune factors, may promote the onset and chronic nature of acne [10,87]. Papular and pustular inflammatory acne lesions tend to be more abundant in *C. acnes* phylotype IA versus in the IB and II phylotypes [10,103], and the involvement of certain loci may not only implicate specific *C. acnes* phylotypes like IA as pathogenic but may also directly relate the presence of such phylotypes to acne severity [10,104]. For instance, *C. acnes* phylotypes have been found to showcase differential expression patterns of proteins and enzymes, such as hydrolases [10]. The presence of pathogenic *C. acnes* strains within the follicle can lead to the release of: (i) inflammatory factors such as porphyrins, butyric acid, propionic acid, TNF-α, lipase, and PAR-2; (ii) virulence factors like hyaluronate lyase, proteases, and matrix metalloproteinases like MMP-13; and (iii) enzymes that target the extracellular matrix (ECM) in the epidermis [10,61,88]. Moreover, free radical production by *C. acnes* can increase inflammation in the pilosebaceous unit and can add to the ability of the follicular epithelium to rupture in the locale of a developing acne lesion [105]. Furthermore, the activation of immune cells, like Th1 and Th17 cells, and the pro-inflammatory factors they produce, such as IL-17, may also be mediated by specific *C. acnes* strains [10]. Thus, the pathogenic activity of *C. acnes* may be characterized by certain pathogenic phylotypes that are resident in the pilosebaceous unit at the time of acne lesion formation [10,103]. While it may be important to understand the taxonomy of *C. acnes* and the role of pathogenic *C. acnes* strains in mediating inflammatory responses, a challenge to this approach arises owing to the fact that several acne-causing and so-called “neutral” (i.e., type IB) *C. acnes* phylotypes may be present together in a single pilosebaceous unit; this adds complexity to therapeutically targeting certain *C. acnes* pathogenic strains in acne [10].

To summarize, *C. acnes* plays an important role in inflammation and virulence in acne pathogenesis [10]. This raises the question as to whether certain pathogenic *C. acnes* strains are sensitive to clindamycin and other topical antibiotic treatments in acne patients and whether targeting specific *C. acnes* strains with novel topical antibiotic or probiotic therapies may be relevant [10,48]. While clindamycin can inhibit the production of lipases formed by *C. acnes* [106], the extent to which clindamycin may also inhibit the range of inflammatory effectors produced by *C. acnes* metabolism or inflammatory signaling during acne pathogenesis requires further research.

### 4.3. Effects of Clindamycin in Treating Inflammatory Lesions

#### 4.3.1. Clindamycin Topicals Reduce Inflammation in Acne

Although *C. acnes* biofilm formation and increasing resistance to topical antibiotic treatment may render the antibacterial properties of clindamycin less effective [10,11,61], evidence suggests that clindamycin may directly exert anti-inflammatory properties in acne lesions [20]. The synergistic effects of a clindamycin/BPO topical combination on inflammatory acne may involve clindamycin hindering PMN chemotaxis and BPO reducing the presence of PMNs, thereby preventing their release of damaging reactive oxygen species (ROS) in the skin and dampening inflammation [20]. Fixed-combination clindamycin/BPO topicals can also mitigate antibacterial resistance to *C. acnes*; the bactericidal nature and potentially mild anti-inflammatory effects of BPO [20] lend additive properties to the bacteriostatic and possible bactericidal characteristics of clindamycin in topical acne treatments [11,14,18]. In addition, the topical retinoid adapalene [107] has been found to reduce TLR-2 expression levels in the skin after treatment with an adapalene topical (i.e., 0.1% adapalene) [90]. Therefore, it is inferred that the incorporation of adapalene into a clindamycin/BPO combination treatment regimen may further hinder inflammation in acne.

Clinically, a clindamycin/BPO topical (i.e., clindamycin 1.2%, BPO 3.75%) can reduce inflammatory acne lesion counts [62]. Of note, the new fixed-combination, triple-combination treatment containing clindamycin, BPO, and adapalene (IDP-126) was found to be effective in reducing the number of inflammatory acne lesions in the treatment of moderate-to-severe acne when compared with all other dyad (i.e., clindamycin/BPO, BPO/adapalene, clindamycin/adapalene) or vehicle treatments tested [13,17,19]. Regarding the entire expanse of acne therapies (i.e., topical antibiotics, BPO, topical retinoids, azelaic acid, and oral tetracycline-class antibiotics), however, oral isotretinoin may still exert the most potent effects over other therapies for the treatment of severe, inflammatory acne [61]. Nevertheless, topical treatments, including fixed-combination clindamycin topicals, have been continuously shown for years to leverage the benefits of cutaneous tolerability, efficacy, and suitability as primary or adjunctive treatments for treating acne across a range of severity [11,13,17,18,19,20,62].

Synergy exists between the anti-inflammatory and antibacterial qualities of clindamycin, thus allocating a crucial role for clindamycin in mitigating the inflammation associated with inflammatory acne, which can often be chronic and disfiguring and can greatly impact the quality of life of acne patients [15,87,92]. Future concerted efforts that aim to investigate the mechanisms of inflammation involved in skin conditions like acne, and the amelioration of inflammation as mediated by clindamycin, will be wholly worthwhile. Novel advances in our understanding of clindamycin’s mechanism of action, the interaction between clindamycin and the skin microbiome [93], and the anti-inflammatory targets of clindamycin may provide insight into the development of potential therapies for several dermatologic conditions with inflammatory etiology or inflammation accompanying infection.

#### 4.3.2. Topical Clindamycin May Subside Inflammation in Mild HS, Rosacea

Clindamycin treats other inflammatory skin conditions in addition to acne, especially those which are papular or pustular in nature, such as HS [34]. HS etiology may largely stem from an autoimmune response unaccompanied by an active infection [35] and is thought to involve blockage of the hair follicles, immune cell recruitment, and ensuing inflammation [34], with roots likely found in the onset and sustained activity of a T helper cell (Th1/Th17) immune response [70]. Systemic propagation of a Th1/Th17 response likely occurs concomitantly with and due to a variety of internal and external influences as outlined above [70]. Blockage of the hair follicle opening in affected regions may provide a logical reason for using clindamycin in mild HS cases [70] as clindamycin targets *C. acnes* within the pilosebaceous unit, which contains the hair follicle [91]. However, increasing severity of HS (i.e., presence of abscesses and risk for scarring) [91] warrants the use of advanced therapies that provide more direct and complete relief of inflammation, and other driving factors of disease presentation, at the lesional site(s).

Topical clindamycin or dapsone for treating cases of mild (Hurley stage I) HS may diminish superficial inflammation in pustular or nodular lesions [34,35]. Meanwhile, an oral treatment regimen is generally warranted to hamper systemic inflammation driving the cutaneous presentation (i.e., nodules, abscesses, and/or draining fistulas) [34] in moderate to severe HS (i.e., Hurley stages II and III). For example, tetracyclines, systemic clindamycin and rifampin in combination, corticosteroids (i.e., prednisolone), and ciclosporin A all possess anti-inflammatory properties and may be used for HS treatment after taking into account disease severity, individual patient histories, and contraindications [34]. Importantly, biologics for the treatment of moderate to severe HS, including secukinumab (IL-17A inhibitor) and adalimumab (anti-TNFα), effectively hamper inflammatory cascades that contribute to immune cell and elevated cytokine level activity in HS lesions [33,70]. Many clinical trials are also currently underway to study the effectiveness of a range of promising biologics, as well as JAK-STAT pathway targets [70], for HS.

In rosacea, dysregulated immune system signaling can mediate inflammation [68]. For example, it is thought that communication between keratinocytes and immune cells, like T helper cells and macrophages, contributes to rosacea symptoms [35]. Specifically, the expression of TLRs such as TLR-2 may become upregulated in keratinocytes due to the presence of the above bacterial and mite species [36,68]. Inflammatory signaling, such as the release of peptides like cathelicidins, may also trigger an inflammatory response [36,68]. The expression levels of Th1 and Th17 T helper cells, synonymous with that of overactive TLR-2 expression, are also upregulated in certain forms of rosacea like PPR [36]. Interestingly, activities of immune cells, like Th17 and Th1 cells and macrophages, can also be increased in acne [87]. Thus, a significant interplay exists between the cutaneous skin microbiome and the innate and adaptive immune systems in rosacea, and clindamycin may target some aspects of rosacea presentation [36,69].

Collectively, the anti-inflammatory properties of clindamycin are reflected in its versatility as an effective treatment option for a range of skin conditions with inflammatory characteristics or etiologies, for which clinical presentation involves inflammatory lesions driven in part by elevated immune system activity.

## 5. Antibiotic Resistance to Clindamycin: Trends and Ways to Overcome

### 5.1. Trends in Resistance Rates Due to Clindamycin Use in Dermatological Practice

Resistance to antibiotics, including topical antibiotics, is an increasing global concern [11]. Threatening our ability to manage severe infections, antibiotic resistance rates continue to increase in response to antibiotic use in treating a range of dermatologic conditions. These rates are also largely indicative of how and what types of antibiotic treatments are prescribed in clinical practice around the world [11,17]. For example, antibiotic resistance rates have drastically increased in the case of acne, which affects nearly 10% of the global population [17]. Clindamycin is widely utilized in fixed-combination topical formulations to treat acne due to its effectiveness and the potential for few side effects during treatment [2,11,105]. There remains debate whether acne is considered a “bacterial infection;” nonetheless, antibiotics, such as clindamycin, may operate by reducing both *C. acnes* growth and *C. acnes*-associated inflammation [11]. However, clindamycin, as well as the macrolide erythromycin, are among the top antibiotics with increasing *C. acnes* resistance due to their topical use [10,17]. Thus, the incidence of *C. acnes* strains that are resistant to both clindamycin and macrolides may be largely due to the frequency of prescribing topical clindamycin treatments for acne [2].

Rates of *C. acnes* resistance to clindamycin were reported to be as high as 90% in some regions in 2016 [93]. In Japan, clindamycin resistance increased from around 20% during 2009–2010 to nearly 45% during 2016–2017 [2]. Just under 20% of *C. acnes* strains may possess clindamycin resistance in acne patients [11], and more than 50% of acne patients harbor one or more clindamycin-resistant *C. acnes* strains [93]. Rates of resistance of *C. acnes* to both macrolides and clindamycin were higher than resistance rates for tetracyclines in Indonesia and other countries with similar temperate climates [105]. Interestingly, acne in Indonesia was found to not only be linked with *C. acnes* presence, but also with *S. epidermidis* and *S. aureus* colonization in the skin [105]. Therefore, it is important to acknowledge that a balance exists between multiple bacterial species in the skin—an equilibrium that exerts a role in acne pathogenesis, the potential for antibiotic cross-resistance, and overall antibiotic treatment response [11,105].

### 5.2. Microbial Mechanisms of Clindamycin Resistance

Several modalities exist with respect to clindamycin resistance. These may be dependent upon the prior use of topical clindamycin to treat a dermatologic condition like acne [11]. Growing evidence suggests that *C. acnes* proliferation alone is not the sole cause of acne and that individuals with *C. acnes* do not possess significantly greater *C. acnes* strain proliferation in their pilosebaceous units than individuals without acne [10]. Nevertheless, the concept of pathogenic or virulent *C. acnes* strains, and the ability of *C. acnes* to form biofilms, may elucidate why particular strains contribute to acne pathogenesis to a greater degree and how clindamycin treatment responsiveness may be dependent both upon the types of *C. acnes* strains and how such strains function in the individual patient [10,15,47].

Prior metagenomics research investigating the taxonomic classification and genomic properties of *C. acnes* revealed that certain strains of *C. acnes* can be found in healthy (i.e., phylotype II and IB strains, and potentially some type III strains) or acneic skin (i.e., phylotype IA-1, IA-2 strains) [10,103,108]. The *C. acnes* phylotype IA strains may be important players in the development of acne lesions, as these strains can harness additional components encoded in their genomes to produce genes with virulent properties [10,108]. In contrast, healthy skin-associated *C. acnes* phylotypes (i.e., phylotype II) may harbor protective genomic factors, like CRISPR/Cas loci, that disallow certain *C. acnes* strains from incorporating virulent genes into their genomes [10]. Meanwhile, phylotype IA-2 not only possesses clindamycin resistance, but has also been linked with the development, or worsening, of moderate-to-severe acne lesions [10]. Conversely, phylotype IA-1 may be present in acne lesions across a range of severity [10]. Resistance to antibiotics like clindamycin may rest within the genetic elements of certain *C. acnes* strains (i.e., phylotype IA strains) [10], which may reduce antibiotic sensitivity and effective treatment response in certain individuals. Resistant *C. acnes* strains are able to proliferate and even persist on the skin even after the course of antibiotic treatment [11,94]. Also, pathogenic *C. acnes* strains may work concomitantly with, or in response to, additional factors, like environmental and immunological signals, to trigger the degree of inflammation and the severity of acne lesions during acne pathogenesis [10]. However, some single-locus sequence typing (SLST) findings have suggested that the distribution of acneic *C. acnes* phylotypes among patients with mild versus severe acne may not be significantly different [10,109]. This argument instead suggests that innate immune signaling may predominate over the effects of certain *C. acnes* phylotypes in inflammatory acne lesions with increasing severity [10,109]. Nevertheless, the interplay between the skin microbiome and its compositional diversity, or the loss of it, are crucial factors to consider as improved treatments are needed to overcome antibiotic resistance [10,108].

### 5.3. Molecular Mechanisms of Clindamycin Resistance

#### 5.3.1. Mutations, Methylation, Protein–Antibiotic Interactions, and Efflux Pumps Confer Resistance to Clindamycin

**Figure 4:**
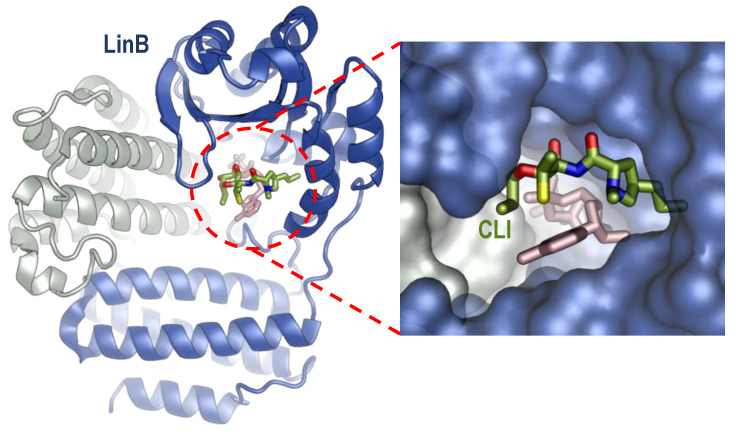
Interaction of clindamycin with the LinB enzyme. Ribbon diagram of LinB depicted on the left. The active site of LinB is occupied by clindamycin (green) and the nonhydrolyzable ATP analog, AMPCPP (beige). One molecule of LinB is shown in blue (surface representation, right); the second copy of LinB is shown in gray. The model in PDB ID 3JZ0 was used to prepare this figure [114]. Nitrogen atoms are blue, and oxygen atoms are red in CLI.

There are three major mechanisms by which bacteria become resistant to the lincosamide class of antibiotics. The first two are based on the alteration of nucleotides of rRNA forming the binding site of the antibiotic, either by a mutation or by the methylation of the nucleotides of the 23S rRNA. Mutations in nucleotide positions 752, 2057–2059, 2452, and 2611 of the 23S rRNA can confer resistance to lincosamides, macrolides, and ketolides (Figure 2) [110]. Resistance to clindamycin is often acquired through *N*^6^-dimethylation of A2058 by erythromycin-resistant rRNA methyltransferases or by C8 methylation of A2503 of 23S rRNA by the Cfr methyltransferase [111,112,113]. The third mechanism of acquired bacterial resistance to lincosamides is through the direct interaction of some proteins with the free antibiotic, which leads to its modification and inactivation, or with the ribosome-bound antibiotic, which evicts the drug from the binding site. For example, enzymes encoded by *lin* genes catalyze adenylylation of lincosamides to inactivate them (Figure 4) [114]. Target-protection proteins, like some ATP-binding cassette (ABC) proteins, can remove the antibiotic from the ribosome to restore translation [115,116]. Lastly, multidrug efflux pumps, including mefA and msrA, may provide another mechanism of resistance to lincosamides like clindamycin, albeit they are more effective in contributing to macrolide rather than lincosamide resistance [117,118].

Mutations in 23S rRNA that affect clindamycin and macrolide sensitivity have been linked to antimicrobial use [119], and the excessive use of clindamycin topicals in treating acne is thought to contribute to the greater incidence of 23S rRNA mutations in *C. acnes* strains [2]. In addition, clindamycin resistance can arise from 23S rRNA methylation [119]. For instance, *N*^6^-dimethylation of A2508 in 23S rRNA can contribute to clindamycin and macrolides cross-resistance at the post-transcriptional stage [14]. Monomethylation of the A2058 residue may also confer lincosamide resistance [40] and thus perhaps clindamycin resistance. The 23S rRNA methylation step also prevents the 7-Cl-MTL of clindamycin from coming into contact with the ribosomal NPET, therefore weakening the binding affinity of clindamycin to the ribosome and promoting clindamycin resistance [3,40]. A mutation at the same adenine residue (A2058G) may also contribute to resistance to clindamycin [38].

#### 5.3.2. Genes Involved in Clindamycin Resistance

On a molecular level, 23S rRNA and 16S rRNA genes in the *C. acnes* genome possess chromosomal point mutations that can give rise to *C. acnes* resistance against macrolides and tetracyclines, respectively [10]. Additionally, the acquisition of the *erm*(X) genes, which give rise to the expression of certain rRNA methyltransferases that catalyze the 23S rRNA methylation step, has been associated with resistance to lincosamide, macrolide, and streptogramin B antibiotics (MLS_B_ cross-resistance) [2,3,10,40]. Importantly, *erm*(X) and *erm*(50) may be significant players in clindamycin resistance [120]. The mono- or dimethylation step essentially prevents certain antibiotics (e.g., lincosamides and macrolides) from binding to the ribosome of the bacterial species of interest, like *C. acnes*, thus initiating *erm*- or *cfr*-mediated antibiotic resistance [3,40].

In addition to the *erm* genes, *cfr* genes, such as that which encodes the rRNA methyltransferase *Cfr*, also play a role in horizontal gene transfer and the acquisition of antibiotic resistance to several antibiotic classes (including lincosamides like clindamycin [113] and some macrolides); this feat can be accomplished via the methylation of the 23S rRNA A2503 residue by Cfr [3]. Resistance to the lincosamides in general may also involve proteins like LsaA—termed target-protection proteins—that can aid bacterial protein translation via binding to the ribosome in place of the antibiotic [3]. Strategies to address target-protection proteins, in addition to *erm*- or *cfr*-mediated antibiotic resistance, could be important routes to investigate in efforts to curtail clindamycin resistance in clinical practice.

#### 5.3.3. Resistance to Clindamycin Involves Multi-Species Crosstalk

The horizontal gene transfer of *erm*(X) between strains of *C. acnes* was found to contribute to the 6-fold greater incidence of resistance in *C. acnes* (*erm*(X) strains) between 2010 and 2015 [2,119]. Importantly, a novel pTZC1 plasmid that harbors both the *erm*(50) gene, which promotes resistance to both clindamycin and macrolides, and the *tet*(W) gene, which leads to resistance to tetracycline, was detected recently in *C. acnes* [119]. Therefore, horizontal gene transfer of the pTZC1 plasmid between strains of *C. acnes* presents one mechanism of acquired resistance to clindamycin [119]. Another study [120] reported that both the *erm*(50) and *tet*(W) genes, especially within *C. acnes* strains of phylotypes IA-1 and IA-2, are increasing in incidence in acne patient samples. Furthermore, transfer of the pTZC1 plasmid may also occur among different bacterial species in the skin; for instance, pTZC1 may be transferred among *C. acnes* and *C. granulosum* strains in acne patients, and the presence of the plasmid may contribute to both clindamycin and macrolide resistance in patient isolates [121]. Thus, *C. acnes* may not be the only player in the emergence of antibiotic resistance in relation to acne. In fact, the idea that antibiotic and multidrug resistance may arise from the transfer of genetic elements between bacterial species on the skin [121] lends urgency to further investigating bacterial strain type and presence, as well as antibiotic use and efficacy, for dermatologic conditions where antibiotic resistance is an imposing threat.

Clindamycin resistance has clinical ramifications outside of the realm of *C. acnes* and acne vulgaris. *S. aureus* resistance to clindamycin can ensue from clindamycin overuse or when clindamycin is a primary treatment for *S. aureus*-related conditions like soft tissue infections or folliculitis decalvans (FD) [2,66]. Interactions between *C. acnes* and *S. epidermidis*—two significant players in the skin microbiome [122]—have been reported with respect to antibiotic resistance stemming from antibiotic use [123]. Strains of both species can be extracted from acne patient samples, and trends in the antibiotic resistance of either species in relation to one another have been reported [123]. For instance, the resistance genes *erm*(A) and *erm*(C) have been found in strains of *S. epidermidis* in acne patient samples; the expression levels of these genes have been associated not only with prior antibiotic or antimicrobial use and *S. epidermidis* resistance, but also with acquired clindamycin and macrolide resistance [123]. Importantly, a fine-tuned balance must exist between species like *S. epidermidis* and *C. acnes* in order to maintain a healthy, homeostatic state in the skin [10] and prevent dysbiosis and ward off inflammation. Given that *S. epidermidis*, like *S. aureus*, plays a role in multidrug resistance, it is important to consider to what extent clindamycin and other antibiotics functionally disrupt the skin microbiome and increase the chances that antibiotic resistance to species like *S. epidermidis* will occur [123].

### 5.4. Assessing Factors Involved in Topical Clindamycin Use and Antibiotic Resistance

Overall, the mechanisms of resistance to clindamycin relate to our current understanding of how clindamycin is prescribed in dermatology. The avoidance of antibiotics as monotherapies is suggested; instead, short-term use of antibiotics is recommended in combination with other treatment modalities, such as BPO or retinoids, like adapalene, to enhance antibiotic effectiveness and prevent antibiotic resistance and excessive *C. acnes* colonization in the skin [10,14,61,124]. In some cases, a BPO/adapalene combination may be a beneficial option to forgo the use of antibiotics altogether [11]. For instance, treatment with a clindamycin topical over a period of 16 weeks led to a 16-fold increase in the incidence of *C. acnes* species that were resistant to the antibiotic [93]. Thus, some resistant *C. acnes* strains may evade primary antibiotic treatment and aid the development of new acne lesions, which is a feat of the bacterium itself that may limit the chances of future antibiotic treatment success, especially if antibiotics are needed or prescribed more than once [93]. Methods to curb antibiotic resistance may involve utilizing antibiotics with narrow-spectrum activity [23,124], perhaps even in topical formulation in the future, along with some of the other treatment types described above. A greater understanding of how *C. acnes* and other bacterial strains may affect clindamycin treatment response and efficacy will be needed in future research [10].

*C. acnes* resistance to antibiotic treatment for acne can be strain-dependent [11], meaning that specific *C. acnes* strains in some individuals may not be fully sensitive to certain antibiotics. However, while some pathogenic and virulent *C. acnes* strains may contribute to antibiotic resistance, such strains may inherently reside in the skin and are not necessarily found to be mutually exclusive in patients who have received prior antibiotic treatment [125]. Therefore, future research may aim to understand how clindamycin can both diminish populous acneic *C. acnes* strains and preserve healthy resident strains in the skin microbiome of acne patients [108], as well as for other dermatology patients for whom topical clindamycin is prescribed. In the United States, dermatologists tend to prescribe more antibiotics for their patients than do other clinicians [61,124]. Therefore, dermatologists have the platform to make key individual and consensus decisions regarding antibiotic use and to recognize antibiotic resistance to clindamycin and other antibiotics as part of the concept of antibiotic stewardship [124]. Resistance to clindamycin and other antibiotics continues to be a significant public health concern [3]; however, clindamycin, when incorporated into fixed-combination topicals, can minimize antibiotic resistance risk and provide successful treatment outcomes [2,13,16,17,18,19,35,59,62].

## 6. The Future Outlook for Clindamycin in Dermatology

Antibiotics have been long-standing treatment options in dermatology for many years, and clindamycin has emerged as a key player in the management of dermatologic conditions like acne and folliculitis. Regarding its success in the management of acne, clindamycin is widely available in fixed-combination topicals with BPO or adapalene, and its efficacy alongside these two actives has been recently evaluated in clinical studies utilizing the novel triple-combination product IDP-126 [13,16,19,126]. Importantly, clindamycin can stunt the growth of *C. acnes* at the same time that it can ameliorate inflammation within the pilosebaceous unit, thus rendering the antibiotic functional against non-inflammatory and inflammatory acne lesions across a range of acne severity and exemplifying its antibacterial and anti-inflammatory properties, respectively [2,18,20]. The potent activity of clindamycin also renders it effective against a number of conditions, such as acute infections of soft tissues [11,36].

Finally, the future for clindamycin in the dermatology space is bright. As insights into the mechanism of action of clindamycin and its anti-inflammatory properties become increasingly elucidated utilizing advances in molecular and structural biology, the effectiveness of novel formulations of clindamycin prescribed in dermatological practice will be enhanced.

## Figures and Tables

**Table 1:** Comparison of the mechanism of action of clindamycin with other antibiotics used for acne vulgaris treatment.

Antibiotic	Class	Mechanism of Protein Synthesis Inhibition	Route of Administration
Clindamycin	Lincosamide	Inhibits the 50S ribosomal subunit at 23S rRNA within the PTC	Mainly topical
Erythromycin	Macrolide	Inhibits 50S at 23S rRNA within the NPET	Mainly topical
Tetracycline, Doxycycline, Minocycline, Sarecycline	Tetracycline	All inhibit 30S at 16S rRNA within the A-site; sarecycline inhibits *C. acnes* ribosome at a second site within the NPET	Mainly oral, minocycline also topical

Adapted from [3,25,37,38,39,40,41,42,43].

## Data Availability

No new data were created or analyzed in this study. Data sharing is not applicable to this article.
